# Enhancement of Measurement Distance in Vision Sensor-Based System Using Super-Resolution for Dynamic Response Measurement Under Low-Illumination Conditions

**DOI:** 10.3390/s26216944

**Published:** 2026-11-03

**Authors:** Junho Gong, Kyuwan Lee, Dooyong Cho

**Affiliations:** 1 Department of Future & Smart Construction Research, Korea Institute of Civil Engineering and Building Technology, Goyang-si 10223, Republic of Korea; jhgong@kict.re.kr; 2 Department of Convergence System Engineering, Chungnam National University, Daejeon 34134, Republic of Korea

**Keywords:** vision sensor, dynamic displacement measurement, low illumination, super-resolution, structural health monitoring

## Abstract

Displacement is a key response index for evaluating the serviceability and safety of civil infrastructure. Although vision sensors have emerged as non-contact and cost-effective alternatives to conventional sensing systems, their performance deteriorates under low-illumination and long-distance conditions because reduced spatial resolution and weakened local intensity information make feature extraction and tracking unreliable. To address this limitation, this study investigates the feasibility of integrating a real-world super-resolution (SR) framework into a vision-sensor-based system for dynamic displacement measurement under low-light conditions. A GAN-based RealSR model was employed to restore low-resolution image sequences, and the restored images were processed using a KAZE-based feature tracking approach. The proposed framework was validated through shaking table tests of a three-story frame structure under a low-illumination condition of 120 lux at measurement distances ranging from 10 to 40 m. Dynamic displacement responses induced by a harmonic excitation of 9.0 Hz were estimated and compared with reference measurements obtained from a linear variable differential transformer (LVDT). The results show that the SR-restored images provided more stable feature extraction and more reliable displacement tracking than bicubic upsampling, particularly for natural targets under low-light and long-range conditions. Furthermore, the application of SR improved the measurability of targets at extended distances, demonstrating that real-world SR is an effective preprocessing strategy for robust vision-based displacement measurement under low illumination.

## 1. Introduction

Structural displacement is one of the most informative response indices for assessing the serviceability, safety, and dynamic characteristics of civil infrastructure [1,2]. Because displacement directly reflects structural deformation and vibration behavior, it has been widely used in structural health monitoring, condition assessment, and modal identification [1,2]. Conventional contact-type sensing systems, such as linear variable differential transformers (LVDTs), accelerometers, and strain-based sensors, have long been employed for displacement-related measurements because of their reliability and well-established sensing principles. However, these systems generally require direct installation on the structure, complex wiring, and continuous maintenance, and in some cases, they also require fixed reference conditions, which may limit their applicability in full-scale or long-term monitoring environments [1,2].

To overcome these limitations, vision-based sensing techniques have been actively investigated as non-contact and cost-effective alternatives for structural displacement measurement [3,4,5]. By analyzing digital image sequences acquired from a remote location, a vision sensor can estimate structural motion without physically attaching transducers to the measured component. Previous studies have shown that such systems can provide sufficiently accurate displacement measurements for bridges, frame structures, and other civil infrastructure, and their applicability has gradually expanded from target-based laboratory validation to target-free and multi-point monitoring in more realistic environments [3,4,5,6]. In particular, vision-based sensing is attractive for long-range applications because it enables structural responses to be monitored in situations where direct access is difficult or where the installation of conventional sensors is impractical [3,4,5].

Despite these advantages, the practical sensing range of a vision sensor is fundamentally constrained by image quality. As the measurement distance increases, the target occupies fewer pixels in the image, resulting in reduced spatial resolution, pixellation, and loss of local texture information [4,5]. These degradations weaken the reliability of feature extraction and tracking, and their influence becomes even more pronounced when natural structural targets are used instead of artificial fiducial markers [5,6]. Accordingly, the effective measurement distance of a vision sensor is governed not only by the optical configuration of the imaging system, but also by whether sufficient structural image information remains available for stable displacement estimation.

This limitation becomes more severe under low-illumination conditions. In dim environments, the acquired images suffer not only from a reduction in pixel density at long distances, but also from decreased brightness, weakened contrast, insufficient local intensity variation, and increased sensor noise [6,7,8]. As a result, structural features that remain distinguishable under normal illumination may become ambiguous or even unrecognizable when the same target is observed under low-light conditions. In this sense, low illumination should not be regarded merely as an environmental factor that reduces tracking accuracy; rather, it acts as a key factor that shortens the usable measurement distance of a vision sensor by accelerating the loss of image features required for stable displacement estimation. This issue is particularly critical in dynamic displacement measurement, where short exposure times are often required to avoid motion blur, thereby further restricting the amount of usable image information.

A number of previous studies have shown that illumination-related image degradation is one of the major obstacles to reliable vision-based structural monitoring. Lee et al. [7] proposed a vision-based displacement measurement method robust to light-induced image degradation for in-service bridges and demonstrated that changes in lighting conditions can substantially affect displacement tracking performance. Lu et al. [8] developed a displacement measurement framework based on deep learning and digital image processing to cope with ambient-light variations and validated its performance under daytime, dusk, and nighttime conditions. Jeong and Jo [6] further reported that variations in luminous conditions, along with occlusion and haze, remain major sources of uncertainty in long-term vision-based displacement monitoring. These studies collectively suggest that illumination is not merely a secondary imaging condition, but one of the dominant parameters governing the practical applicability of vision-based sensing in field environments.

Nevertheless, many previous studies addressing illumination variation or dim environments have focused primarily on improving robustness at the detection or tracking stage through illumination-tolerant tracking schemes, deep-learning-based detectors, or auxiliary target design [6,7,8]. Although such approaches are useful in specific situations, they do not fundamentally resolve the problem that the input image itself may already be degraded to a level unsuitable for long-range displacement measurement. When low illumination and long measurement distance occur simultaneously, image degradation is compounded by both reduced spatial resolution and weakened local contrast. Under such conditions, even a robust tracking framework may exhibit limited performance because the visual information required for stable tracking has already been severely diminished. Therefore, extending the effective measurement distance in low-light environments requires not only robust tracking algorithms, but also a strategy for restoring degraded input images before feature extraction and motion estimation.

From this perspective, image super-resolution (SR) offers a promising approach to addressing the coupled limitations of distance and illumination. SR aims to reconstruct a higher-resolution image from a low-resolution input and can potentially recover boundaries, textures, and locally meaningful details that are beneficial for feature extraction and displacement tracking [9,10]. Recent deep-learning-based SR models have shown strong capability in restoring images degraded by realistic blur and noise, particularly when they are designed for real-world rather than idealized degradation conditions [9,10]. In the context of structural monitoring, the significance of SR lies not simply in improving visual appearance, but in recovering structural image information that can support more reliable sensing when the original acquisition conditions are unfavorable.

Although SR-assisted extension of measurement distance has been preliminarily discussed under normal indoor illumination [11], it remains unclear whether the same strategy is equally effective under low-light conditions, where the degradation associated with increasing distance is compounded by contrast loss and noise amplification. This distinction is important because a method that performs well in a bright and stable environment may not necessarily retain its usefulness when the sensing range is further restricted by adverse illumination. Accordingly, the present study focuses specifically on whether a real-world super-resolution framework can recover the usable measurement distance of a vision sensor for dynamic displacement measurement under dim conditions.

Therefore, this study investigates whether the effective measurement distance of a vision sensor under low-light conditions can be extended by incorporating a real-world SR model into a feature-point-based dynamic displacement measurement framework. To this end, two representative feature extraction strategies—the Shi–Tomasi corner detector and the KAZE feature detector—are comparatively examined in order to clarify how the benefit of SR varies with the type of features used for tracking [12,13,14,15]. The proposed framework is validated through shaking table tests conducted under normal- and low-illumination conditions, and its effectiveness is assessed in terms of image quality, feature-point stability, and displacement measurement performance as the measurement distance increases.

The contributions of this study can be summarized as follows. First, the coupled effects of low illumination and increasing measurement distance on vision-based dynamic displacement measurement are quantitatively investigated from the perspectives of spatial resolution loss, feature ambiguity, and local intensity degradation [6,7,8]. Second, a real-world super-resolution framework is introduced as a preprocessing strategy prior to feature extraction and tracking, and its capability to recover the usable sensing range under low-light conditions is experimentally evaluated [9,10,11]. Third, by comparing the traditional corner-based baseline represented by Shi–Tomasi with the degradation-robust nonlinear feature detector KAZE, the detector-dependent characteristics of SR-assisted distance extension are systematically examined [12,13,14,15]. Through this approach, the present study aims not only to verify whether SR is effective under dim conditions, but also to clarify how its practical benefit depends on the subsequent feature-tracking strategy.

In recent years, super-resolution has begun to be explored as a means of relaxing the resolution limit of vision-based displacement measurement. Sun et al. [16] enhanced the displacement measurement accuracy by applying a GAN-based super-resolution model to low-resolution images, and Wu et al. [17] combined deep-learning-based super-resolution with feature-coordinator preservation to improve the accuracy of vision-based measurement. Similar strategies have been adopted in adjacent structural monitoring tasks, such as automated crack detectability in in situ bridges [18] and concrete crack detection [19], in which super-resolution served as a preprocessing step for recovering high-frequency image details. In parallel, a separate line of research has addressed the influence of illumination: Lee et al. [7] and Lu et al. [8] developed displacement measurement schemes robust to light-induced image degradation, Zhai et al. [20] introduced an iterative Otsu segmentation scheme for LED targets under low-light conditions, and Ngeljaratan and Moustafa [21] enhanced underexposed images to recover trackable features. Recent reviews of low-light image enhancement [22] and of vision-based displacement measurement [23] provide a broader context for these efforts. However, the two directions have largely been pursued independently. Super-resolution studies have been conducted mainly under normal indoor illumination [11,16,17,24], whereas low-light studies have relied on illumination-tolerant detectors, auxiliary target design, or image enhancement rather than on restoring the spatial resolution of the input image [7,8,20,21]. Consequently, it remains unclear whether super-resolution is effective when the reduction in spatial resolution and the loss of local intensity information occur simultaneously, as they do in dim, long-range field measurements. In addition, recent work has shown that tracking performance can be improved by combining conventional trackers with learned models [25,26] or by fusing vision with other sensing modalities [27]; these approaches, however, do not address the degradation of the input image itself.

The novelty of the present study lies in addressing this gap. First, whereas the earlier feasibility study by the present authors [11] examined super-resolution only under normal indoor illumination, the present work explicitly couples low illumination with increasing measurement distance and quantifies how the two degradation mechanisms interact. Second, super-resolution is treated as a preprocessing stage whose benefit is judged by measurement performance rather than by image appearance, and its effect is separated from that of simple interpolation by comparing original, bicubic, and real-world super-resolution images within an identical measurement pipeline. Third, by comparatively evaluating a conventional corner detector (Shi–Tomasi) and a nonlinear-scale detector (KAZE) [28], the study shows that the benefit of super-resolution is detector-dependent, a dependency that has not been reported previously. Fourth, the results demonstrate that super-resolution can convert measurement conditions that are practically unusable in the original low-light images into usable data for natural targets, thereby extending the effective measurement range under poor illumination. Taken together, these aspects position the present work not as another application of super-resolution to displacement measurement, but as a systematic investigation of whether, and under which conditions, real-world super-resolution recovers the sensing capability of a vision sensor in low-light, long-range monitoring.

It should also be noted that GAN-based super-resolution may synthesize high-frequency structures that are not physically present, and such hallucinated details could in principle produce apparently stable but incorrect feature trajectories [29,30]. This issue is therefore treated as a methodological concern and discussed in Section 4.

## 2. Materials and Methods

### 2.1. Problem Definition and Overall Framework

The central question addressed in this study is whether real-world super-resolution can recover or extend the practical measurement distance of a vision sensor for dynamic displacement measurement under low-light conditions. To answer this question, a vision-based displacement measurement framework was established in which degraded image sequences are first restored and then introduced into a conventional feature-point-based tracking pipeline. The key premise is that the sensing capability of a vision system is determined not solely by camera hardware or imaging geometry, but by the distance and illumination conditions under which sufficient feature information can still be stably extracted and tracked. Under low-light conditions, the target appears smaller, darker, and less distinguishable in the image, and the usable sensing range is therefore expected to decrease more rapidly than under normal illumination. In this study, SR is thus treated not merely as a visual enhancement tool, but as a preprocessing strategy intended to improve the measurement utility of the input image before feature detection and tracking.

The overall procedure consists of four stages. First, image sequences of the vibrating structure are acquired at different measurement distances under normal- and low-illumination conditions. Second, three image sets are prepared from the acquired frames: the original images, bicubic interpolation images, and real-world SR-restored images. Third, structural feature points are extracted from predefined regions of interest (ROIs), and their motion is tracked over time to estimate pixel-level displacement. Finally, the image-based displacement is converted into physical displacement and compared with reference sensor data. Through this framework, the influence of SR on the practical distance limit of the vision sensor can be systematically evaluated for different feature extraction strategies [3,11,12,13,14,15].

### 2.2. Vision-Based Dynamic Displacement Estimation

**Figure 1:**
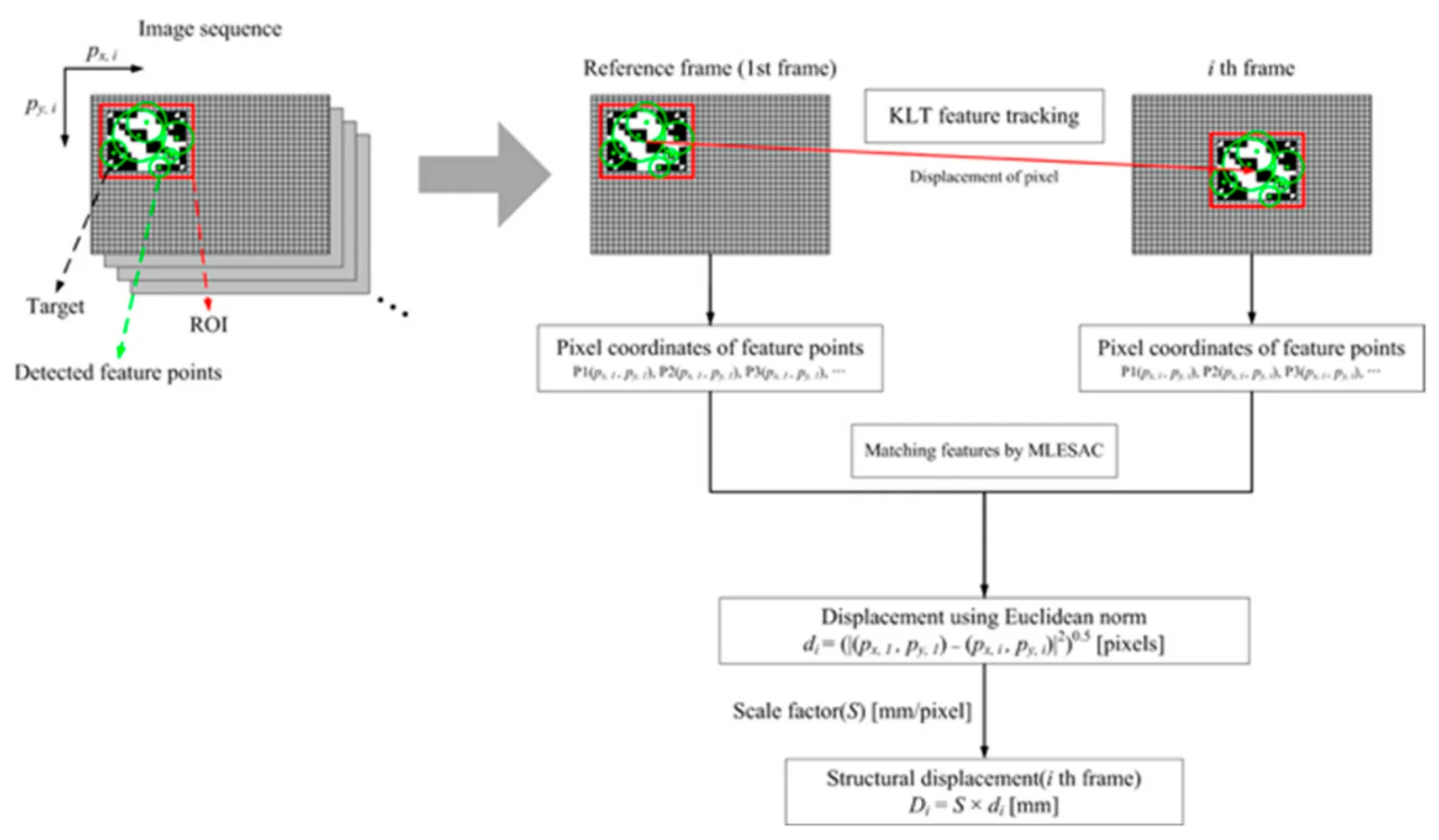
Feature points based structural displacement measurement system.

The dynamic displacement of the structure was estimated using the feature-point-based vision measurement system shown in Figure 1. In this framework, the first frame of each image sequence was used as the reference frame, and ROIs were defined on the structural components to be monitored. Feature points within the selected ROIs were extracted and then tracked frame by frame to determine the motion trajectories of the structural target. The pixel-level displacement was subsequently converted into physical displacement using a scale factor derived from the imaging geometry and the known dimensions of the target region. This procedure has been widely adopted in previous vision-based structural displacement studies because it enables non-contact structural response estimation directly from remotely acquired image sequences [3,4,5,6].

In the present study, two feature extraction schemes were comparatively examined. The first was the Shi–Tomasi corner detector, which identifies corner-type image features based on local gradient information [12]. Shi–Tomasi is a representative baseline detector that is computationally efficient and highly effective when the target contains distinct corner patterns with sufficiently strong intensity variation. However, its detectability may deteriorate rapidly when the target has limited corner information or weak contrast, as is often the case for natural structural surfaces. The second was the KAZE feature detector, which constructs a nonlinear scale space and can preserve meaningful local structures under degraded image conditions more effectively than conventional linear-scale detectors [13]. The two detectors were selected not because one was assumed to be universally superior, but because they represent two different feature extraction philosophies: a traditional corner-based baseline and a nonlinear feature detector expected to be more robust under degraded imaging conditions. Their comparative use makes it possible to examine whether the effect of SR-assisted distance extension depends on the detector characteristics.

After feature extraction, the detected points were tracked over time using the Kanade–Lucas–Tomasi (KLT) tracking algorithm [14]. Because low-light image sequences are prone to unstable or mismatched correspondences caused by noise, weak texture, and local blur, an outlier rejection step based on Maximum Likelihood Estimation Sample Consensus (MLESAC) was introduced [15]. Only inlier feature points satisfying the dominant geometric transformation were retained for the final displacement estimation. This process was adopted to reduce the influence of erroneous matches and unstable trajectories on the measured responses, particularly under degraded imaging conditions. It should be noted that the present study does not propose a new tracking algorithm. Rather, it investigates how restoration of the input image affects the actual measurement performance and practical distance limits within a consistent feature-tracking framework. Accordingly, the methodological emphasis is placed on clarifying the interaction between image restoration and feature-based displacement estimation, rather than on developing a new motion-tracking formulation.

For a consistent comparison, the same ROIs and the same tracking parameters were applied to all image sets. Within each region of interest, multiple feature points were detected and tracked, and the displacement reported in this study is the average over those feature points; the same procedure was applied to the original, bicubic and super-resolved images. Corner points were detected within the predefined ROIs and tracked using MATLAB R2020a. PointTracker (Kanade–Lucas–Tomasi) implementation was with its default settings: a 31 × 31 pixel neighborhood, a three-level image pyramid, and a maximum of 30 iterations per point. The forward–backward error threshold was left at its default value (Inf), so the tracker did not discard points on the basis of bidirectional error and the MLESAC step described above was solely responsible for outlier rejection. The built-in sub-pixel refinement of the tracker was used; this implementation does not expose the interpolation kernel as a user-settable parameter. Because the physical area covered by the tracking window depends on the image resolution, the resolution of the three image sets is stated here. In the feasibility study of Section 3.2, the original, bicubic, and super-resolved images were all resampled to the same 1080 × 1920 pixel resolution, so the tracking window covered the same physical area in all three sets. In the long-range study of Section 3.3, the super-resolved images were generated at twice this resolution (2160 × 3840 pixels), so the same pixel window covered half the physical area. The contribution of image restoration, as distinct from that of simple upscaling, was assessed through the resolution-controlled comparison of the bicubic and RealSR images in Section 3.2.

### 2.3. Real-World Super-Resolution for Low-Light Range Recovery

To alleviate image degradation under long-distance and low-illumination conditions, a real-world super-resolution framework was introduced prior to feature extraction and tracking. The role of SR in the present study is not simply to generate enlarged images, but to recover structural boundaries, local texture, and trackable visual details that are insufficiently represented in the original low-light, long-range frames. If such structural information can be restored to a level suitable for stable tracking, displacement measurement may become feasible even at distances approaching or exceeding the practical limit of the original image sequence.

Among the various SR approaches that have recently been developed, GAN-based real-world super-resolution models are particularly relevant to practical engineering images because actual image degradation rarely arises from ideal bicubic downsampling alone. Instead, real images typically contain composite degradation factors, including blur, noise, compression artifacts, and sensor-related distortions [9,10]. In this study, a RealSR-type framework that reflects such realistic degradation characteristics was employed on the premise that it would be better suited to restoring the boundary and texture information required for feature detection in structural images. This approach is also consistent with the general direction of recent GAN-based SR research, which emphasizes the perceptually meaningful recovery of high-frequency structures rather than simple interpolation-based enlargement [9,10].

The super-resolution model adopted in this study is the publicly released pretrained real-world SR model of Ji et al. [10], which estimates blur kernels and injects realistic noise in order to reproduce practical degradation. The model is the official PyTorch version 1.0 implementation released by the authors at https://github.com/jixiaozhong/RealSR (accessed on 7 August 2026) and is built on the BasicSR framework; it was the winning entry of the CVPR NTIRE 2020 Challenge on Real-World Super-Resolution. No additional training or fine-tuning on the present dataset was performed; the pretrained weights released with the implementation were applied as provided. Two sets of restored images were generated. In the feasibility study of Section 3.2, the original frames were downsampled by a factor of 0.25, that is, to 270 × 480 pixels, and were then upscaled by a factor of 4 using either bicubic interpolation (BC) or the RealSR model (RSR), so that the restored images were returned to the same 1080 × 1920 pixel resolution as the original (Org.) images and all three sets could be compared at an identical pixel resolution. In the long-range study of Section 3.3, the super-resolved images were generated at 2160 × 3840 pixels, that is, at twice the resolution of the original frames, in order to exploit a finer pixel grid at extended measurement distances.

For the SR-assisted analysis, the original frames acquired at each measurement distance were processed using both bicubic interpolation and RealSR-restored imaging. The resulting image sets were then analyzed using the same ROIs, the same feature detection procedures, and the same tracking and outlier rejection processes. By comparing the original, bicubic, and RealSR conditions within an identical measurement pipeline, the effect of SR on the recovery of trackable structural information and on the extension of the measurable distance under low-light, long-range conditions could be directly evaluated. In other words, the purpose of this section is not to assess whether SR produces visually more attractive images, but to determine whether it can restore the measurement-relevant structural information that is insufficient in the original image and thereby improve the practical effectiveness of feature-based displacement estimation.

### 2.4. Experimental Program

**Figure 2:**
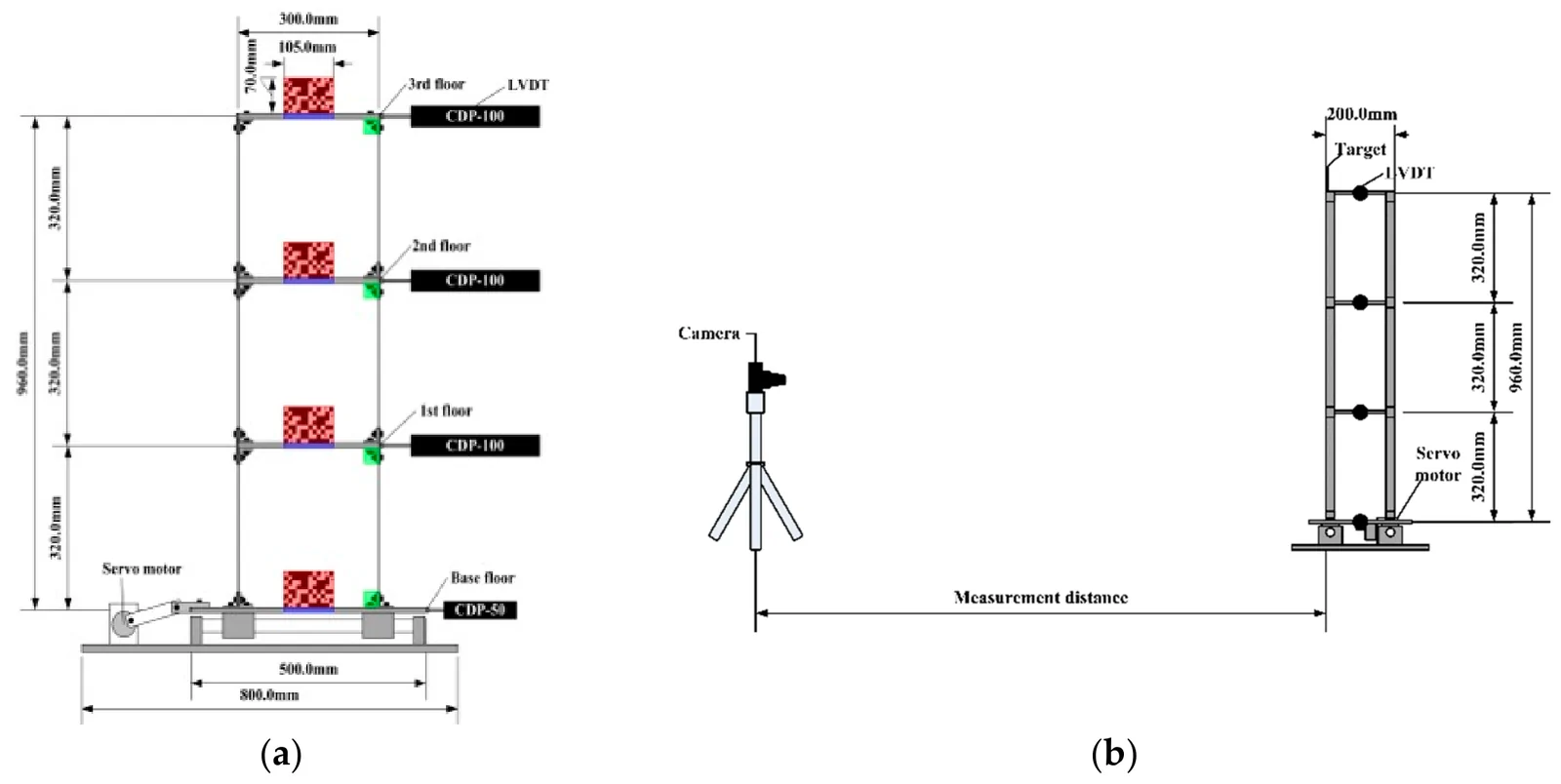
Indoor experimental configuration. (**a**) Frame structure model. (**b**) Shaking table test setup (side view).

To validate the proposed framework, indoor shaking table tests were conducted using a three-story frame structure, as shown in Figure 2. The structural responses were simultaneously measured using a vision sensor and reference displacement sensors, and the experimental program was designed not only to compare displacement accuracy but also to evaluate the practical measurement distance limit under different illumination conditions. Unlike many previous laboratory validation studies performed mainly under stable lighting conditions, the present experiments explicitly included a low-illumination environment in order to systematically examine the reduction in usable measurement distance [7,8].

Both artificial and natural targets were considered in the experimental program. The artificial target was a fiducial marker of approximately 105 mm × 70 mm, attached at the midpoint of each slab (red regions in Figure 2a). The natural targets consisted of bolt shafts (5 mm and 10 mm in diameter), nuts, and slab cross-sections of approximately 105 mm × 10 mm (green regions and blue regions in Figure 2a respectively). This target configuration was intended to represent both marker-installed scenarios and practical marker-free structural monitoring situations in which existing structural components must be used as measurement targets. The normal-illumination condition was defined as the baseline case for establishing the reference sensing range of the vision sensor, whereas the low-illumination condition was introduced to reproduce the image degradation commonly encountered in practical monitoring situations, including reduced brightness, weakened contrast, and insufficient local intensity variation.

A Sony DSC-RX100M7 (Sony Corporation, Tokyo, Japan) camera was used for image acquisition, and the focal length was set to 72 mm (equivalent to 200 mm in the 35 mm format). The camera was installed at a stationary location and aligned with the structure using a laptop for remote control of the recording conditions. The structure was subjected to horizontal harmonic excitation at 9.0 Hz with an amplitude of ±14.5 mm, thereby generating stable dynamic displacement responses. Image sequences were recorded for 180 s at a resolution of 1080 × 1920 pixels and a frame rate of 120 FPS.

To ensure reproducibility and to exclude the influence of automatic exposure control, the camera settings were fixed throughout all measurements: aperture f/4.5, shutter speed 1/125 s, ISO 800, exposure compensation 0 EV, average metering, the built-in flash disabled, the exposure program set to manual, and the white balance fixed at 3200 K. The sensitivity was determined by the fixed ISO 800 setting, and no separate gain adjustment was applied. The identical settings were applied under both the normal-illumination (450 lux) and low-illumination (120 lux) conditions so that the difference in the recorded image intensity originated solely from the reduction in illuminance and not from a change in exposure. For the harmonic excitation at 9.0 Hz with an amplitude of +/−14.5 mm, the peak target velocity is approximately 820 mm/s; the corresponding target displacement within a single exposure (1/125 s) is about 6.6 mm, which is equivalent to approximately 6.9, 3.4, 2.3, and 1.7 pixels at measurement distances of 10, 20, 30, and 40 m, respectively. This residual motion blur, together with the sensor noise amplified at ISO 800, is regarded as a limiting factor at short measurement distances.

**Figure 3:**
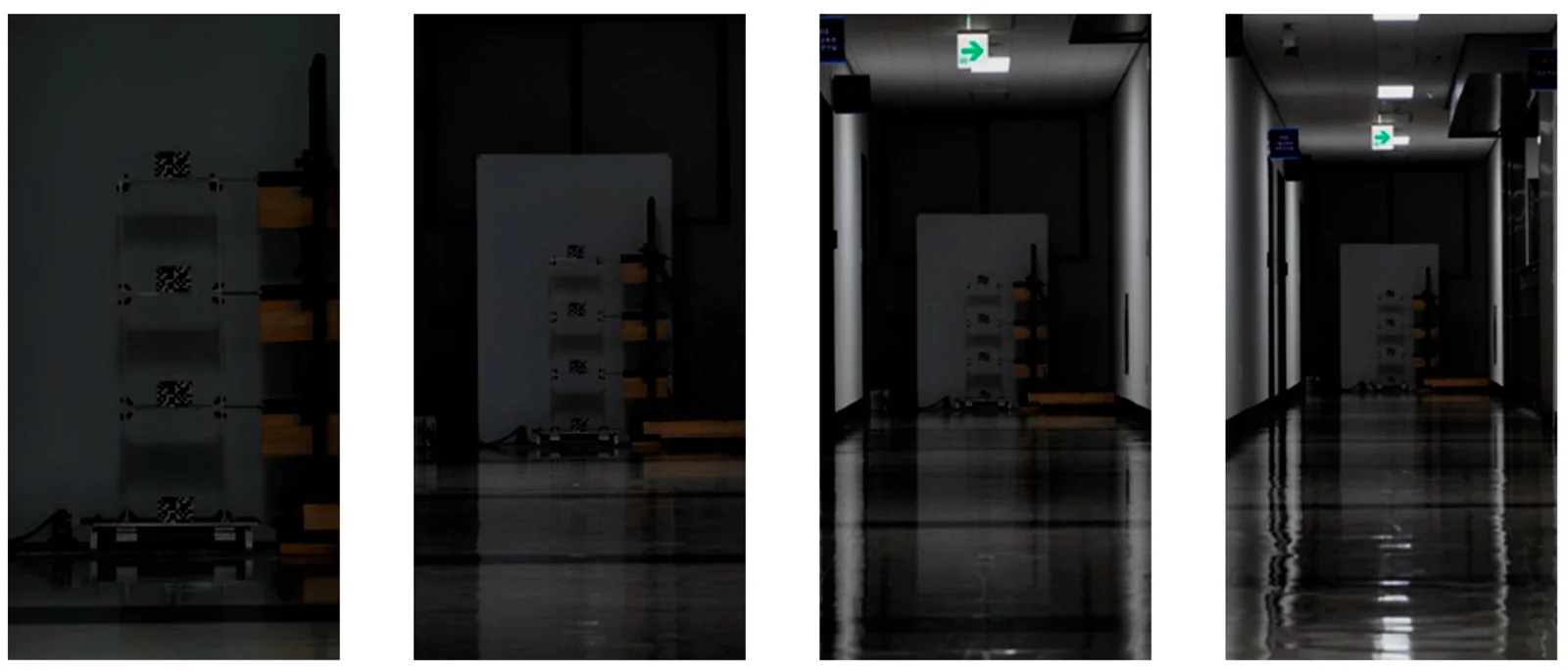
Field of view of frame structure in low illumination. Measurement distance ranging from 10 m (**left**) to 40 m (**right**) with 10 m increments.

The measurement distance was increased from 10 m to 40 m in increments of 10 m. The scale factor at 10 m was 0.95 mm/pixel, and it increased to 1.91 mm/pixel, 2.87 mm/pixel, and 3.82 mm/pixel at 20 m, 30 m, and 40 m, respectively. This means that the same physical displacement is represented by progressively fewer pixels as the measurement distance increases. The illumination level under the normal-illumination condition was maintained at 450 lux, whereas that under the low-illumination condition was maintained at 120 lux throughout the experiments (Figure 3). By varying both distance and illumination in this manner, the experimental design enabled the coupled effects of spatial resolution reduction and local contrast degradation on vision-based displacement measurement to be systematically examined.

The motivation for this design is that illumination and distance do not affect the measurement system independently, but in a coupled manner. At short distances, measurement may still remain feasible even under dim conditions because the target occupies a sufficient number of pixels. However, as the observation distance increases, the number of pixels representing the target decreases rapidly, and the adverse effect of low illumination becomes increasingly amplified. Accordingly, the experiments were organized so that the measurement distance was progressively increased under a fixed illumination level, and the displacement estimation performance obtained from the original, bicubic, and super-resolved image sequences was compared at each distance. This arrangement made it possible to determine whether SR could recover the practical usable sensing range of the vision sensor under low-light, long-range conditions.

### 2.5. Performance Evaluation Metrics

The performance of the proposed framework was evaluated from two perspectives: image restoration quality and displacement measurement accuracy. Image quality was assessed using PSNR, SSIM, and LPIPS [31,32]. Among these, PSNR and SSIM are full-reference metrics that compare a restored image with a reference image, whereas LPIPS is a perceptual similarity metric that is more closely aligned with human visual perception [31,32]. In this study, a predefined reference image was assigned for each target, and the restored images were quantitatively evaluated against that reference in order to assess their structural fidelity and perceptual quality. This comparison made it possible to distinguish the visual and structural differences between simple interpolation-based enlargement and RealSR-based restoration.

However, the ultimate objective of the present study is not image enhancement itself, but the extent to which such restoration improves the actual displacement measurement performance. Therefore, the image quality metrics were not treated as independent end-point criteria, but rather as complementary indicators interpreted in conjunction with feature detectability and tracking stability. Displacement errors were evaluated by comparing the estimated responses with the reference LVDT measurements using the root mean square error (RMSE) and the relative peak error (RPE). RMSE represents the average error level over the entire time history, whereas RPE reflects the accuracy of amplitude reproduction. By comparing RMSE and RPE across illumination conditions, measurement distances, target types, and image restoration strategies, the overall performance of the vision-sensor-based displacement measurement system was comprehensively assessed [3,4,5,6,11].

The relative peak error is defined as $\mathrm{RPE}=100\times \frac{d_{v,\mathrm{peak}}-d_{L,\mathrm{peak}}}{d_{L,\mathrm{peak}}}$, where *d_v_*_,*peak*_ and *d_L_*_,*peak*_ denote the peak amplitudes of the vision-based and the LVDT responses, respectively. The sign is retained: a positive RPE indicates that the vision-based measurement overestimates the peak amplitude, whereas a negative RPE indicates an underestimation, and the magnitude represents the relative error. In the present experiments, negative RPE values were obtained for the natural targets at long measurement distances under low illumination, where the reduced number of usable feature points caused the tracked displacement amplitude to be underestimated. The application of super-resolution reduced this underestimation and shifted the RPE toward small positive values.

## 3. Results and Discussion

### 3.1. Baseline Measurement Performance Under Low Illumination and Comparison with Normal Illumination

This study primarily focuses on the displacement measurement performance of the vision sensor under low-illumination conditions and compares the results with those previously reported under normal illumination. To this end, feature detection and displacement measurement were performed using one artificial target and two natural targets. The artificial target was a fiducial marker, whereas the natural targets consisted of a bolt connection and a slab cross-section. Two feature detection schemes, namely the Shi–Tomasi corner detector and the KAZE feature detector, were adopted to extract representative key points from each target region. For brevity, the displacement responses obtained from the Shi–Tomasi- and KAZE-based tracking approaches are hereafter referred to as STC and KZF, respectively. The objective of this section is to examine how low illumination, together with increasing measurement distance, affects feature detectability and displacement measurement accuracy, and to clarify how these trends differ from those observed under normal illumination.

**Figure 4:**
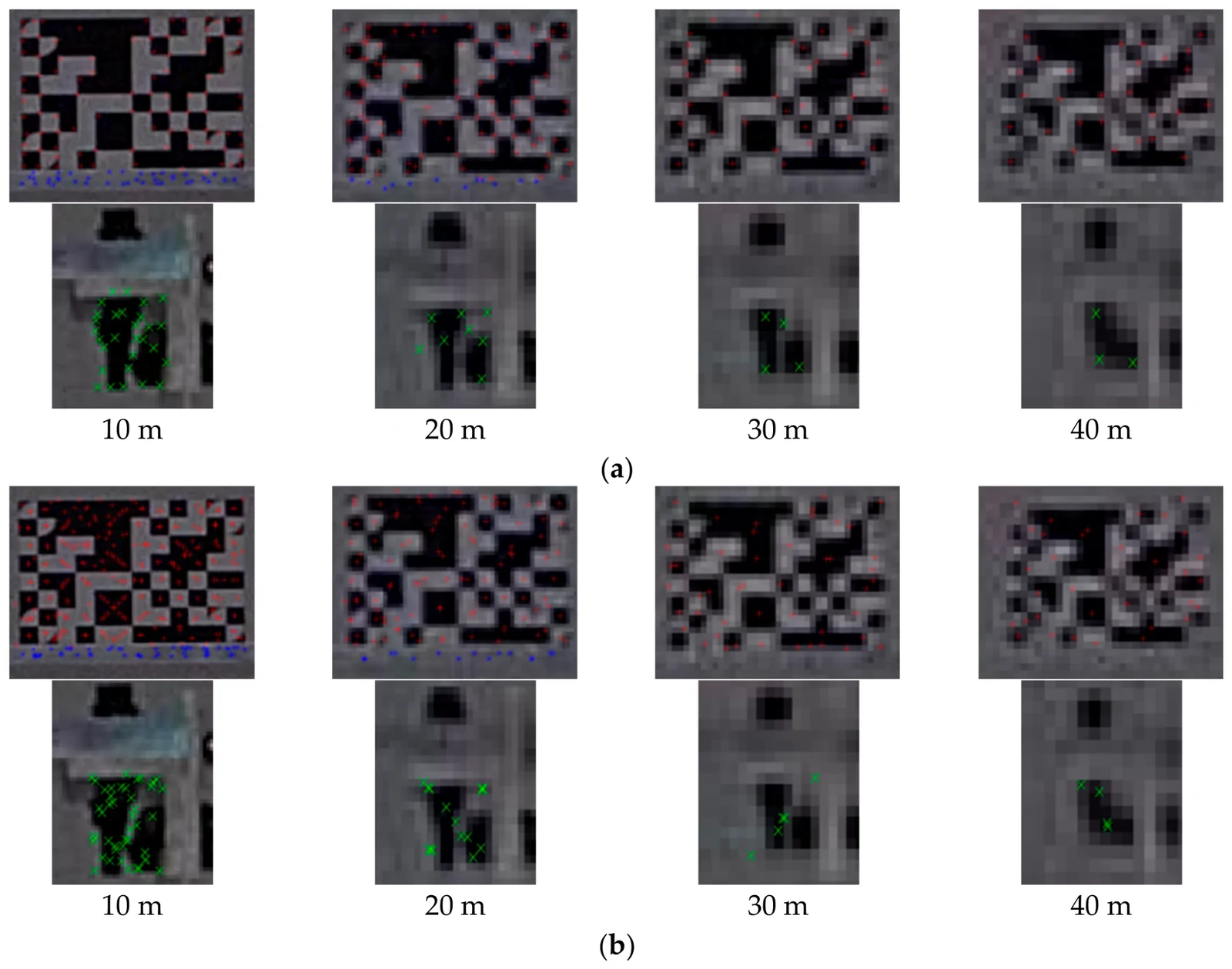
Feature detection results depending on measurement distance changes. Red +, blue * and green × mean the detected points within the fiducial target, slab cross-section and bolt connection respectively. (**a**) Corner point detection. (**b**) KAZE feature detection.

Figure 4 presents representative feature detection results obtained under different measurement distances and illumination conditions. As the measurement distance increased, the effective number of pixels representing the target region gradually decreased, and this tendency became more critical under low illumination because of reduced brightness, increased image noise, and the loss of local texture information. The artificial target maintained relatively stable feature visibility owing to its regular geometric pattern and high contrast. In contrast, the natural targets exhibited much greater sensitivity to both distance and illumination because their detectable features depended on irregular surface texture and local edge information rather than intentionally designed markers. In addition, the KAZE detector generally retained a larger number of valid feature points than the Shi–Tomasi detector, indicating that the former was more robust to scale reduction, blurred boundaries, and illumination-induced texture degradation.

The quantitative feature detection results are summarized in Table 1. For the artificial target under normal illumination, the number of feature points detected by the Shi–Tomasi detector was 90, 82, 41, and 29 at 10, 20, 30, and 40 m, respectively, whereas the corresponding numbers for the KAZE detector were 288, 100, 54, and 34. Under low illumination, the Shi–Tomasi detector identified 101, 100, 39, and 28 feature points, while the KAZE detector identified 289, 99, 46, and 31 feature points. These results indicate that for the artificial target, the dominant source of deterioration in feature detectability was the increase in measurement distance rather than illumination alone, although low illumination further reduced the stability of the extracted features at longer distances. For the bolt-connection target, the number of detected feature points was substantially smaller than that of the artificial target. Under normal illumination, the Shi–Tomasi detector identified 25, 9, 4, and 2 feature points from 10 to 40 m, whereas the KAZE detector identified 36, 16, 10, and 5 points. Under low illumination, the Shi–Tomasi detector identified 29, 8, 3, and 2 points, while the KAZE detector identified 35, 14, 8, and 5 points. This confirms that the KAZE detector provided more stable feature detection than the Shi–Tomasi detector even for natural targets with limited texture information. The slab cross-section showed the most severe deterioration among the three target types. Although more than 45 feature points were detected at 10 m, the number of valid points decreased sharply to approximately 9–16 at 20 m, and feature detection became nearly infeasible at 30 m and 40 m regardless of the detector. These results demonstrate that the baseline detectability of the vision sensor is governed not only by illumination and distance, but also by the intrinsic geometric and textural characteristics of the target.

**Figure 5:**
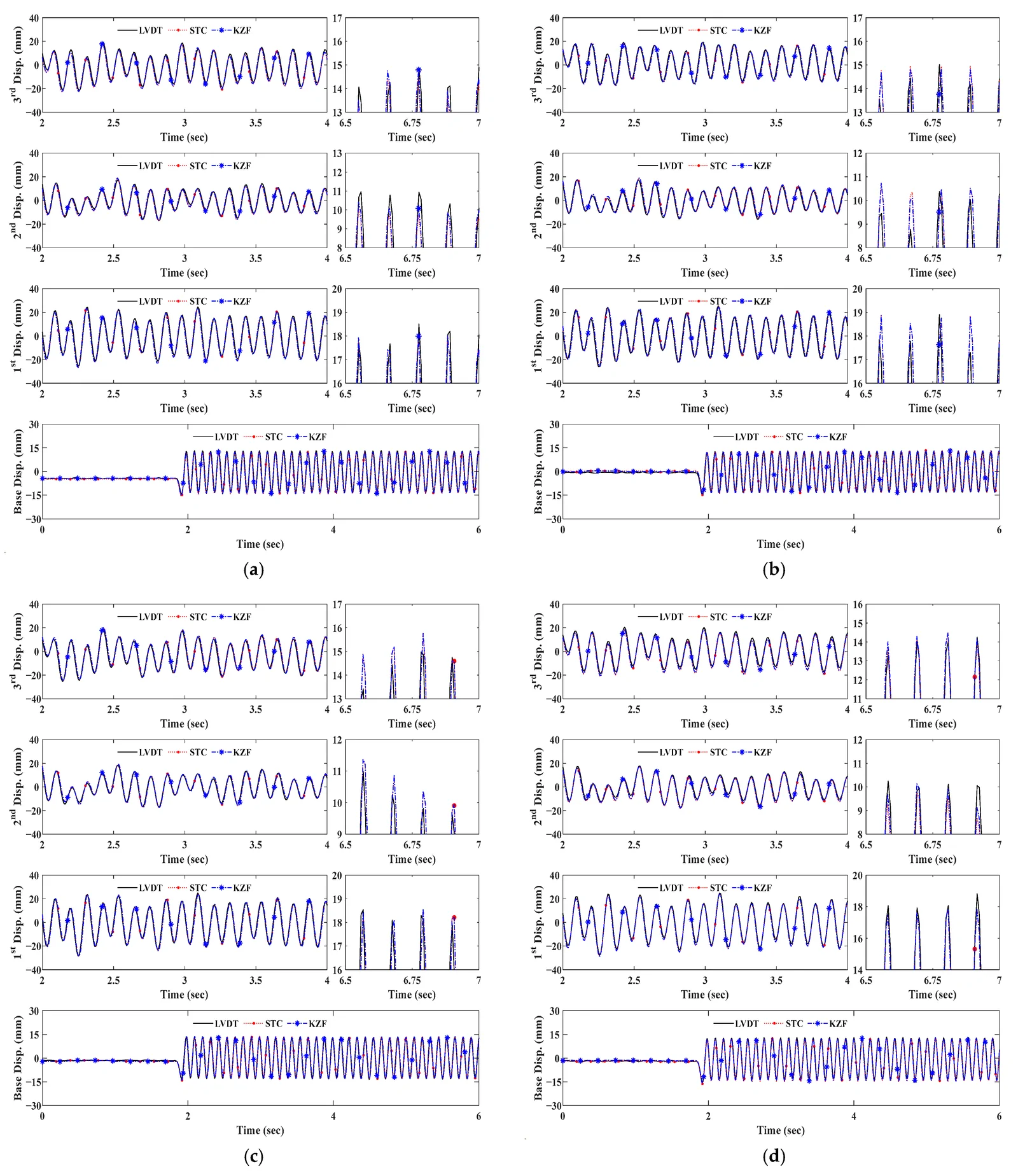
Displacement measurement result comparison of fiducial target. (**a**) Measurement distance of 10 m. (**b**) Measurement distance of 20 m. (**c**) Measurement distance of 30 m. (**d**) Measurement distance of 40 m. All cases in this figure were acquired under low illumination (120 lux).

**Figure 6:**
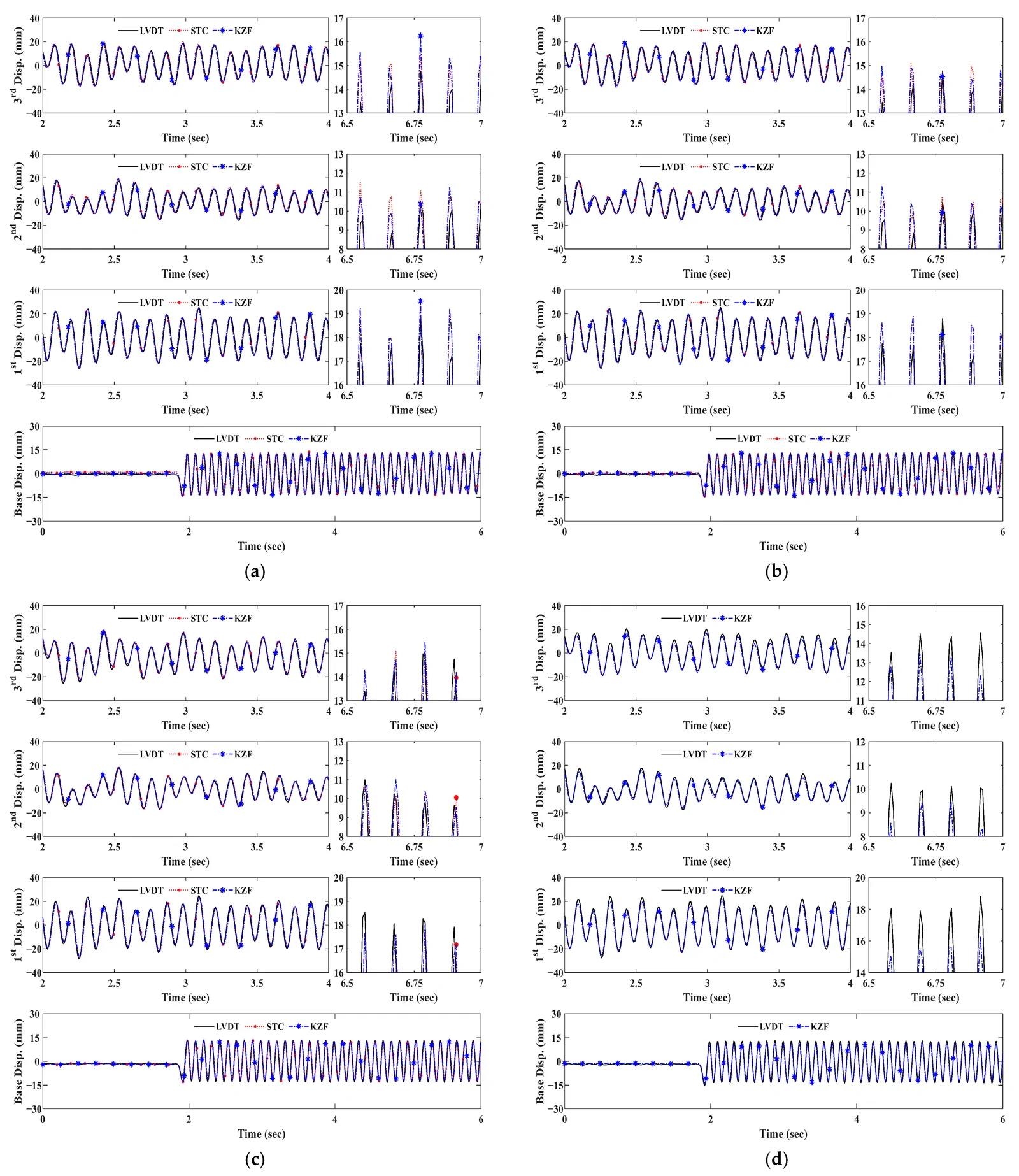
Displacement measurement result comparison of bolt connection. (**a**) Measurement distance of 10 m. (**b**) Measurement distance of 20 m. (**c**) Measurement distance of 30 m. (**d**) Measurement distance of 40 m. All cases in this figure were acquired under low illumination (120 lux).

**Figure 7:**
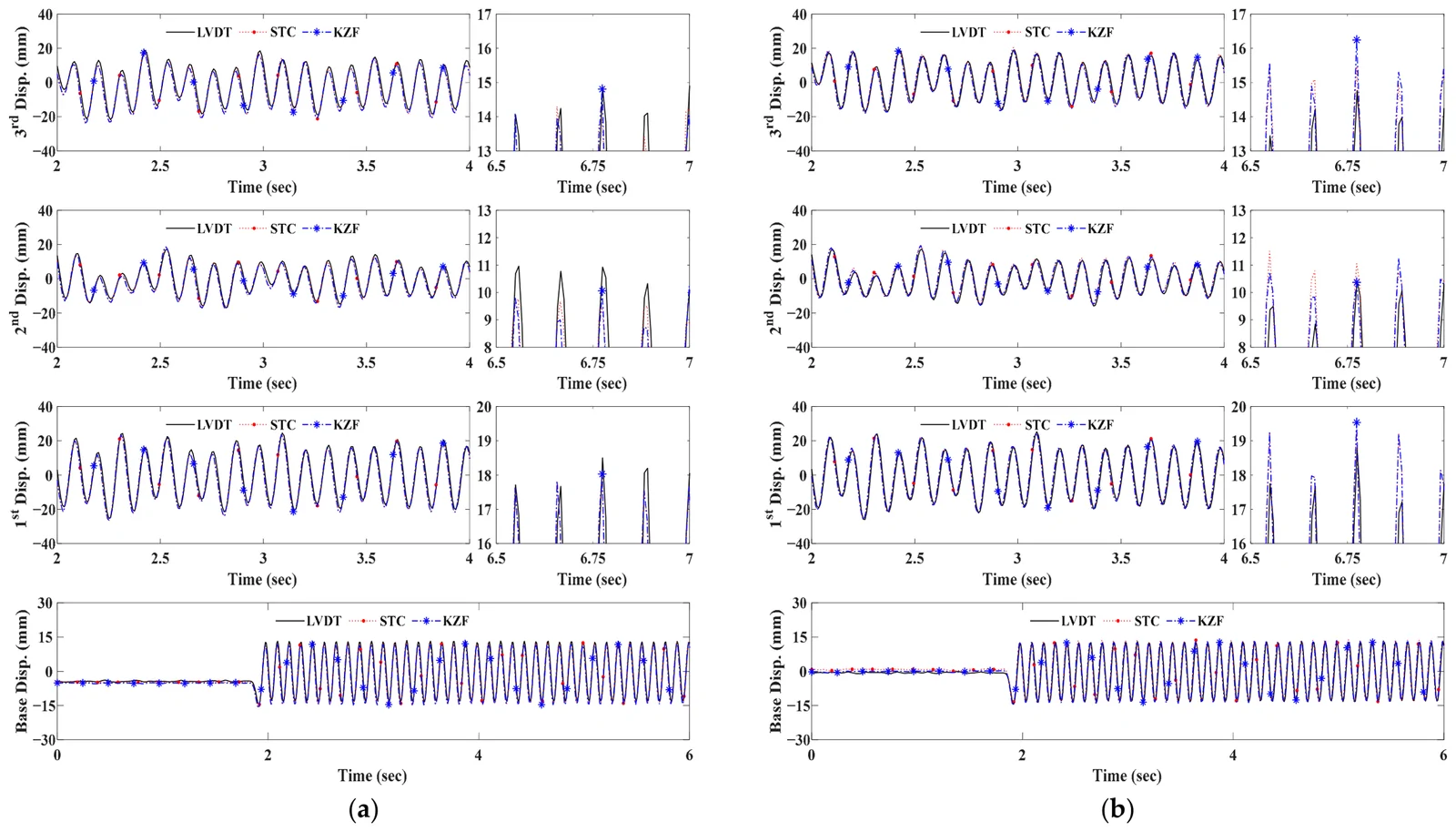
Displacement measurement result comparison of slab cross-section. (**a**) Measurement distance of 10 m. (**b**) Measurement distance of 20 m All cases in this figure were acquired under low illumination (120 lux).

After evaluating the feature detection characteristics, the time-domain displacement measurement performance was examined using the detected feature points. The floor displacement responses estimated from the Shi–Tomasi- and KAZE-based tracking results were compared with the reference measurements acquired from the linear variable differential transformer (LVDT), and the associated measurement errors were quantified in terms of the root mean square error (RMSE) and the relative peak error (RPE). Figure 5, Figure 6 and Figure 7 present representative time-history comparisons for the artificial target, bolt connection, and slab cross-section under low illumination, whereas the normal-illumination results reported in the previous study were used as benchmarks for interpretation.

Figure 5 shows the displacement responses measured from the artificial target under low illumination. At relatively short distances, both STC and KZF reproduced the overall waveform and peak response of the LVDT data with acceptable agreement, indicating that the artificial target still provided sufficiently distinctive features for reliable tracking under moderate low-light conditions. However, as the measurement distance increased, the discrepancy between the vision-based measurements and the LVDT reference gradually increased, reflecting the combined influence of reduced spatial resolution and illumination degradation. In comparison with the previously reported normal-illumination results, the low-light measurements exhibited a more rapid loss of tracking stability at longer distances, even for the artificial target.

Figure 6 presents the displacement measurement results obtained from the bolt-connection target under low illumination. Unlike the artificial target, the bolt connection does not contain an intentionally designed high-contrast pattern and therefore relies on naturally occurring edges and surface texture. As a result, the degradation caused by low illumination was more pronounced, particularly at longer distances. The STC-based responses showed larger deviations from the LVDT reference as the measurement distance increased, whereas KZF maintained comparatively better tracking consistency. Nevertheless, the signal quality of both methods deteriorated as low illumination and long-distance imaging were combined. Relative to the normal-illumination benchmark, the bolt-connection target exhibited a clearer reduction in measurement performance under low illumination, confirming the vulnerability of natural targets to contrast loss and texture weakening.

A similar but more severe tendency was observed for the slab cross-section, as shown in Figure 7. Because the slab surface provides weak and spatially non-uniform texture, its displacement measurement performance deteriorated more rapidly than that of the bolt-connection target. Although the dominant displacement pattern could still be captured at shorter distances, reliable feature extraction and stable tracking became increasingly difficult as the measurement distance increased. This behavior became particularly critical under low illumination, where the slab cross-section approached the practical measurement limit of the vision sensor. In comparison with the previously published normal-illumination results, distances that remained marginally measurable under normal illumination became substantially less reliable under low illumination, highlighting the compounded effect of poor lighting and low spatial resolution.

The qualitative observations in Figure 5, Figure 6 and Figure 7 are quantitatively summarized in Table 2. For the artificial target, the RMSE of the STC-based measurement under normal illumination increased from 0.62 mm at 10 m to 0.75 mm at 40 m, corresponding to an increase of approximately 21.0%. Under low illumination, the corresponding RMSE increased from 0.64 mm to 0.86 mm, corresponding to a larger increase of approximately 34.4%. This comparison clearly shows that the deterioration in measurement accuracy became more pronounced when low illumination and long measurement distance acted simultaneously. A similar trend was observed in RPE. More importantly, the natural targets exhibited a faster rate of error growth than the artificial target, and in several long-distance low-light cases, valid measurements could not be obtained reliably because the number of usable feature points became insufficient for stable tracking. Overall, KZF yielded lower error values and more stable measurements than STC in most test conditions, indicating that the KAZE-based approach is more suitable for low-light, long-range vision-based displacement measurement.

From these results, it can be concluded that the baseline performance of the vision sensor is strongly constrained by illumination condition, measurement distance, and target type. Although the artificial target preserved relatively stable performance over a wider range of conditions, the natural targets exhibited a substantial decline in both feature detectability and displacement accuracy as the imaging condition became more challenging. The discrepancy between normal and low illumination also became increasingly evident with increasing distance. These findings provide the motivation for introducing super-resolution-based image enhancement in the following sections.

**Figure 8:**
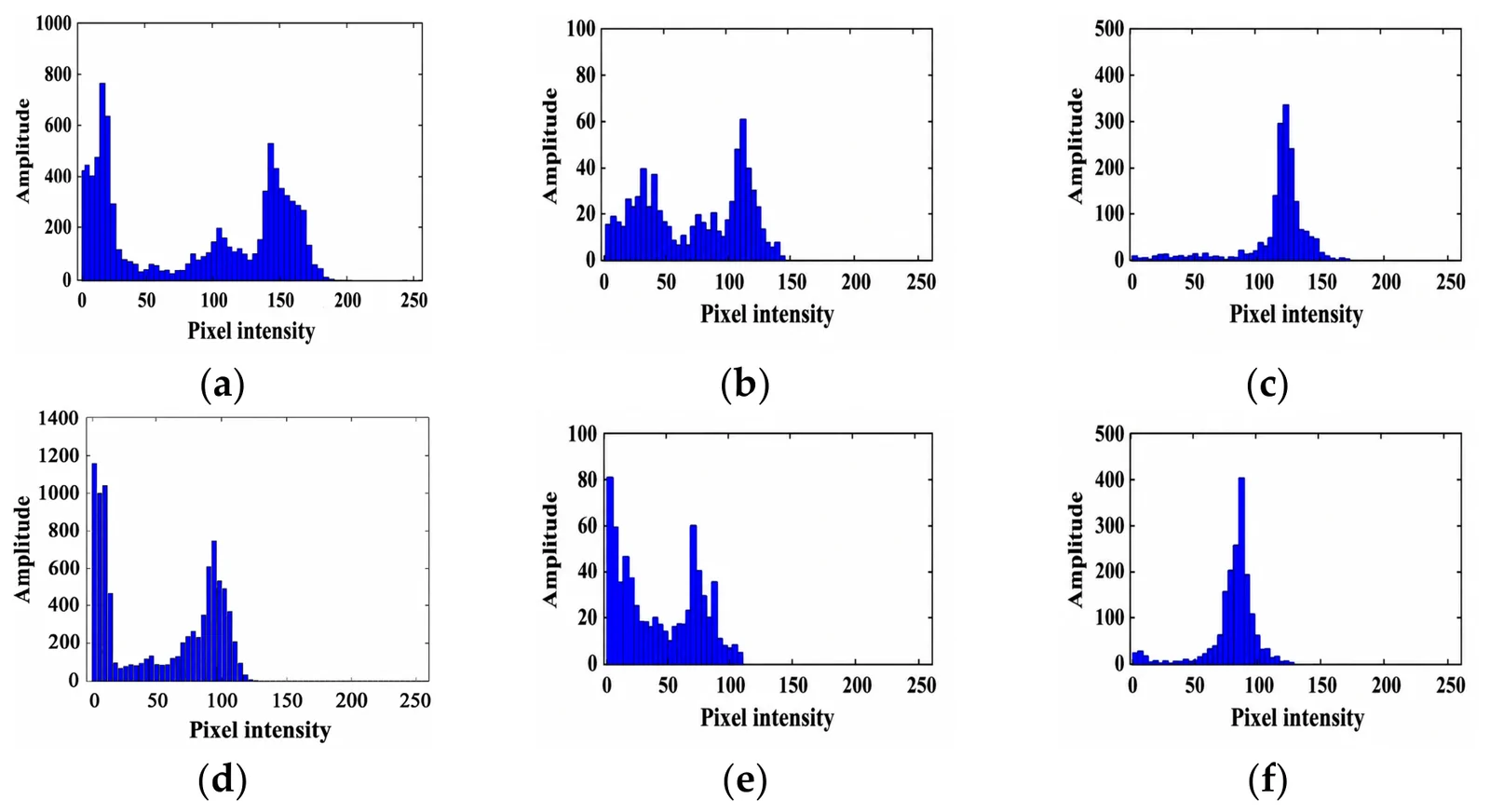
Intensity histograms of the three regions of interest at a measurement distance of 10 m (**a**) Artificial target under normal (450 lux) illumination. (**b**) Bolt connection under normal (450 lux) illumination. (**c**) Slab cross-section under normal (450 lux) illumination. (**d**) Artificial target under low (120 lux) illumination. (**e**) Bolt connection under low (120 lux) illumination. (**f**) Slab cross-section under low (120 lux) illumination. The mean pixel intensity and the RMS contrast are given in each panel.

To characterize the two illumination conditions objectively, the intensity distributions of the three regions of interest were analysed at a measurement distance of 10 m, as shown in Figure 8. Under low illumination the mean pixel intensity decreased to approximately 61–69% of its normal-illumination value (from 86.4 to 54.0 for the artificial target, from 71.8 to 44.0 for the bolt connection, and from 116.7 to 80.7 for the slab cross-section), and the RMS contrast decreased simultaneously in all three regions. Because the camera settings were identical under both conditions, the reduction in both brightness and contrast is attributable to the decrease in illuminance alone. The broadening of the distributions toward low intensity values also indicates that the loss of local contrast, rather than a change in exposure, is the origin of the degradation observed in the subsequent measurement results.

### 3.2. Feasibility Study of Super-Resolution-Based Image Restoration Under Low Illumination

As shown in Section 3.1, the principal causes of performance degradation under low illumination are the loss of structurally meaningful image information due to reduced brightness, increased noise, blurred boundaries, and insufficient spatial resolution. To mitigate these limitations, the feasibility of applying super-resolution (SR) as an image restoration strategy was investigated. Specifically, the original low-resolution image (Org.) was compared with images reconstructed using bicubic interpolation (BC) and the GAN-based RealSR model (RSR). The purpose of this comparison was not merely to assess visual enhancement, but to determine whether the restored structural information could improve feature extraction and displacement measurement under low-illumination conditions.

**Figure 9:**
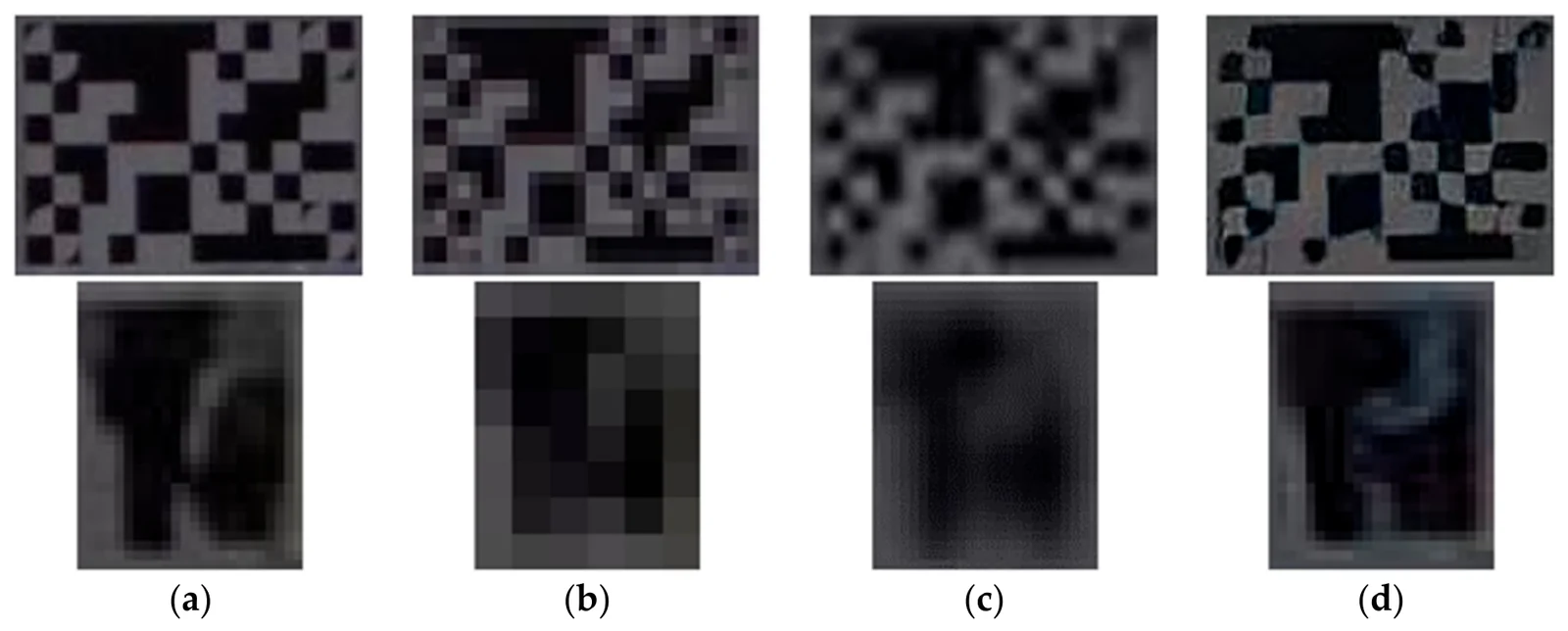
Visualization of upscaled images: (**a**) Org. (1.0). (**b**) BC (0.25). (**c**) BC (1.0). (**d**) RealSR (1.0). The scale factor in parentheses denotes the resolution relative to the original image: 1.0 corresponds to the original 1080 × 1920 pixel resolution, and 0.25 denotes the downsampled input from which the bicubic image was produced. The original (Org.) images have a resolution of 1080 × 1920 pixels, and the bicubic (BC) and RealSR (RSR) images were obtained by downsampling the original frames by a factor of 0.25 and then upscaling them by a factor of 4 back to the same 1080 × 1920 pixel resolution, so that all three images were compared at an identical scale.

Figure 9 shows representative image restoration results obtained using the three image sets. The BC image provided a simple magnified version of the original input; however, the restored image remained relatively smooth and blurred around boundaries and textured regions. In contrast, the RSR image exhibited sharper local edges and more distinguishable structural patterns, particularly near corners, boundaries, and textured surfaces that are critical for feature detection. These visual differences suggest that the effectiveness of SR should be evaluated not only by conventional image fidelity metrics, but also by its usefulness for subsequent measurement tasks.

The quantitative image quality comparison is presented in Table 3. At 10 m under normal illumination, the PSNR values of BC and RSR were 32.11 and 32.34 dB, respectively, while the corresponding LPIPS values were 0.29 and 0.21. Thus, RSR yielded not only slightly higher pixel-level fidelity but also substantially better perceptual quality than BC at this distance. Under low illumination at 10 m, the LPIPS value decreased from 0.33 for BC to 0.27 for RSR, corresponding to an improvement of approximately 18.2%. However, PSNR did not always favor RSR. For example, at 20 m under low illumination, BC yielded a PSNR of 40.70 dB, whereas RSR yielded 36.22 dB. This discrepancy indicates that conventional reference-based metrics such as PSNR and SSIM do not necessarily reflect the suitability of a restored image for displacement measurement. From the perspective of vision-based tracking, the preservation of meaningful structural boundaries and local texture is more important than pixel-wise similarity alone.

**Figure 10:**
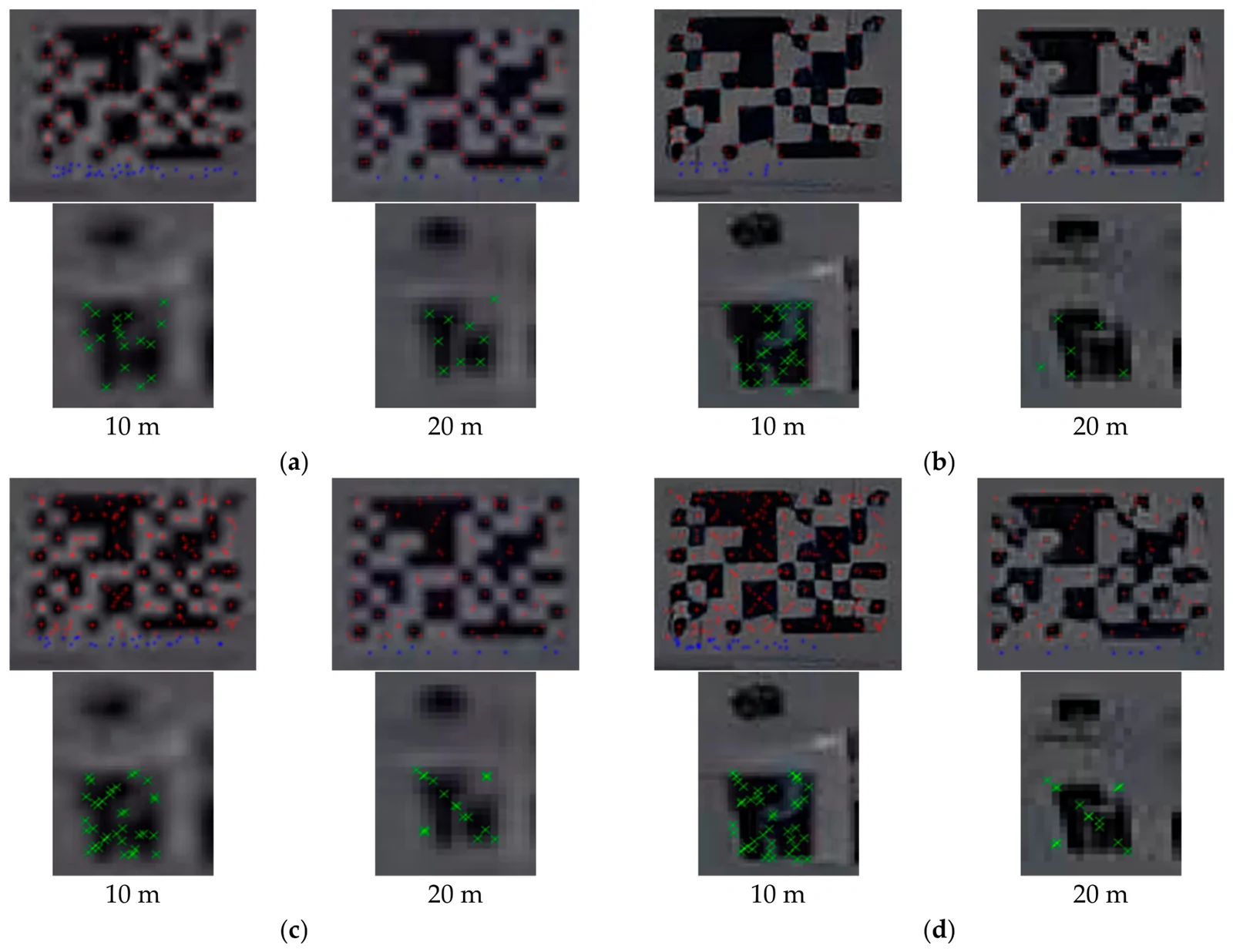
Feature detection results of bicubic upsampled and RealSR images at 10 m and 20 m. Red +, blue * and green × mean the detected points within the fiducial target, slab cross-section, and bolt connection, respectively. (**a**) Corner point detection (BC images). (**b**) Corner point detection (RSR images). (**c**) KAZE feature detection (BC images). (**d**) KAZE feature detection (RSR images).

To verify whether the restored images improved practical tracking performance, feature detection and displacement measurement were repeated using the BC and RSR images. As shown in Figure 10, the RSR images provided clearer and more spatially separable feature points than the BC images, particularly in low-light cases where the original images suffered from blurred boundaries and reduced contrast. This tendency was more evident for natural targets because their usable features were already limited in the original images. Therefore, the role of RSR is not simply to enlarge the image, but to restore target-specific structural information that can support more reliable feature extraction and tracking.

The corresponding displacement measurement results are summarized in Table 4. Under low illumination at 10 m, the RMSE of the KZF-based measurement decreased from 0.64 mm in the original image to 0.58 mm in the RSR image, corresponding to an improvement of approximately 9.4%. At 20 m under low illumination, the RMSE further decreased from 0.73 mm to 0.59 mm, corresponding to an improvement of approximately 19.2%. These results indicate that the benefit of RSR became more pronounced as the low-light imaging condition became more challenging. In contrast, BC showed only limited and inconsistent improvement because simple interpolation enlarged the image without sufficiently restoring the structural information required for accurate feature localization. In addition, KZF generally outperformed STC after SR application, indicating that the KAZE-based approach was more capable of exploiting the restored multiscale texture information provided by RSR.

Overall, the results demonstrate that SR-based image restoration is a feasible preprocessing strategy for low-light vision-based displacement measurement. Even when improvements in PSNR or SSIM were not uniform, the RSR images consistently provided more useful structural information for feature detection and tracking than the original or BC images. Accordingly, the effectiveness of SR should be interpreted primarily in terms of measurement performance rather than image appearance alone. Based on these findings, the following section evaluates whether SR can also alleviate the severe performance degradation observed at longer measurement distances under low illumination.

### 3.3. Enhancement of Long-Range Measurement Performance Under Low Illumination Using Super-Resolution

After confirming the feasibility of SR under low illumination, its effectiveness in extending the usable measurement range was further investigated. In the previously reported normal-illumination experiments, the measurement accuracy for natural targets deteriorated markedly at longer distances because the target region occupied only a limited number of pixels. Under low illumination, this limitation becomes even more critical because the reduction in spatial resolution is accompanied by additional noise, contrast loss, and the degradation of local texture. Therefore, this section focuses on whether SR can recover sufficient structural information to improve long-range displacement measurement under low illumination.

**Figure 11:**
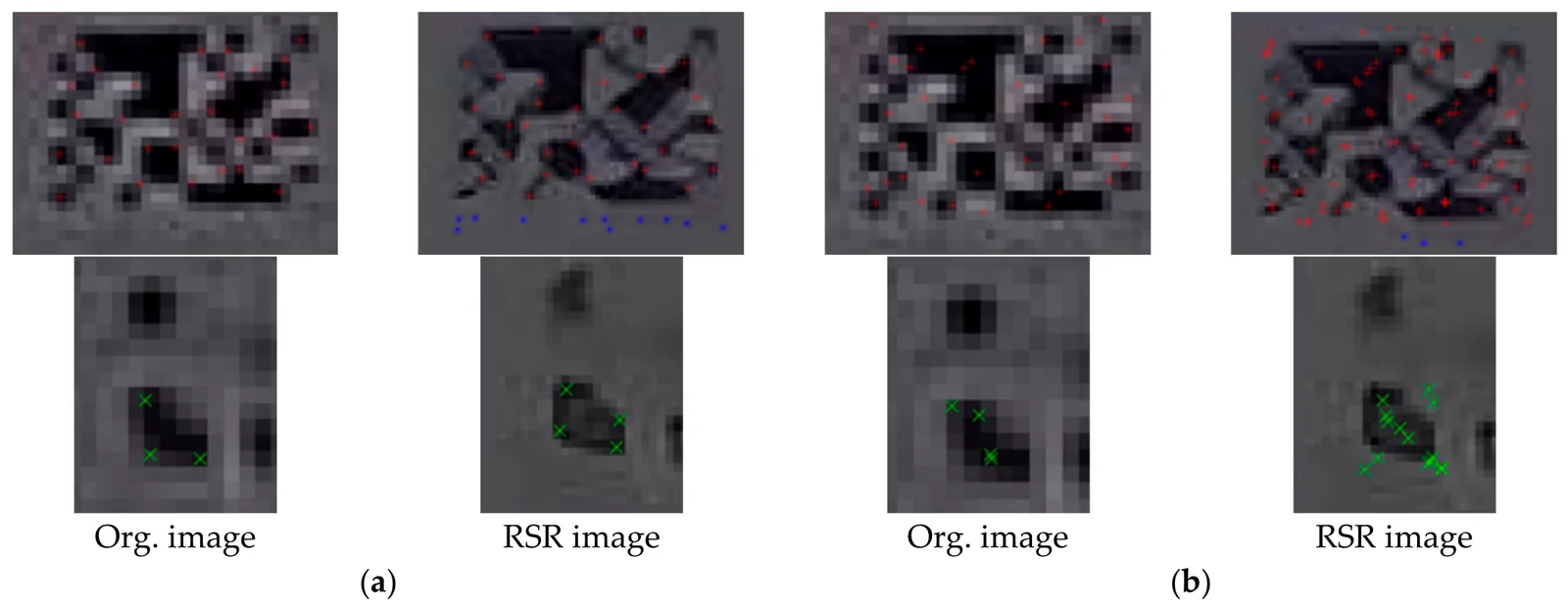
Feature detection results for RealSR images at 40 m. Red +, blue * and green × mean the detected points within the fiducial target, slab cross-section, and bolt connection, respectively. (**a**) Corner point detection. (**b**) KAZE feature detection.

Figure 11 compares the feature detection results obtained from the original and SR-restored images at long measurement distances. In the original low-light images, the number of valid feature points decreased sharply with increasing distance, and the remaining features often appeared weak, clustered, or unstable. In contrast, the SR-restored images provided clearer boundaries and more distinguishable local structures, allowing the Shi–Tomasi and KAZE detectors to identify a larger number of meaningful feature points. This effect was particularly important for natural targets, for which the original low-light images frequently failed to provide enough reliable features for continuous displacement tracking.

**Figure 12:**
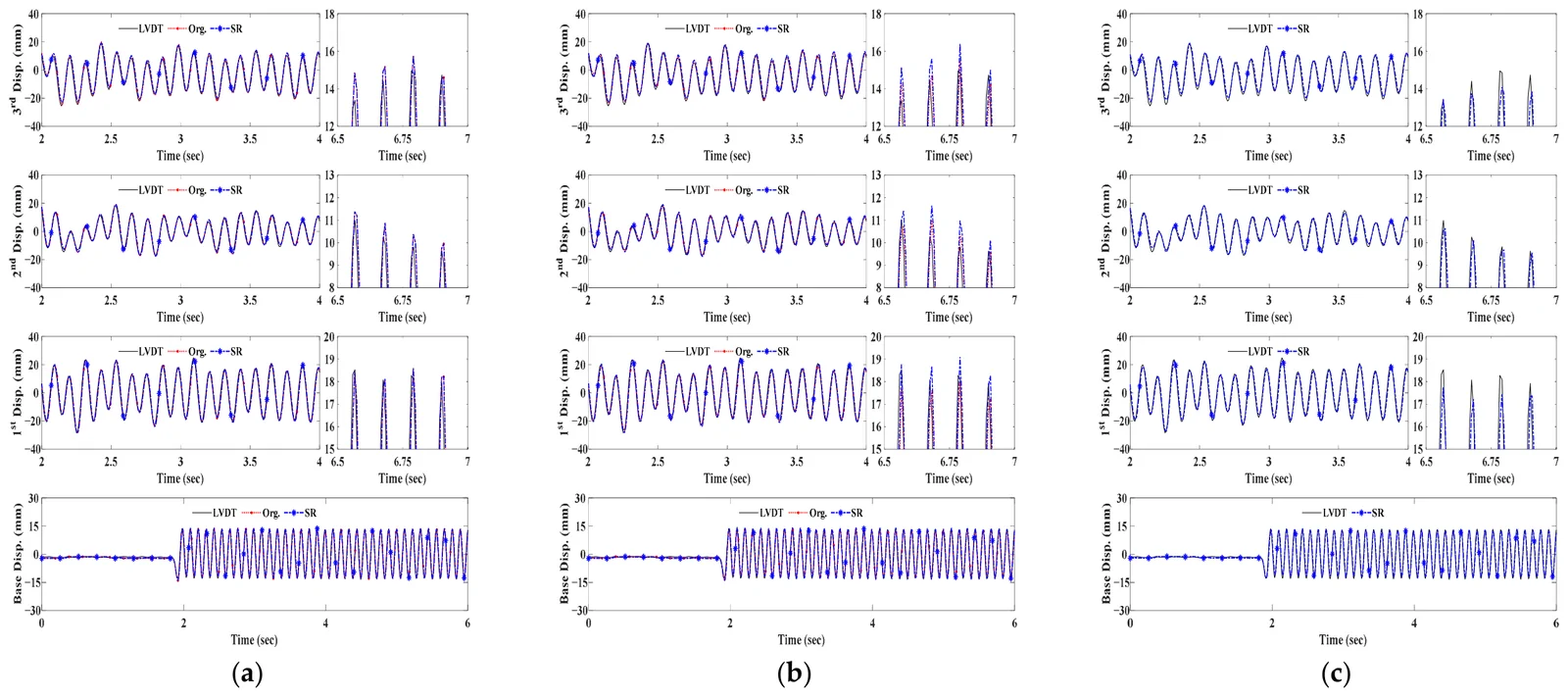
KZF comparison at 30 m depending on targets. (**a**) Artificial target. (**b**) Bolt connection. (**c**) Slab cross-section.

**Figure 13:**
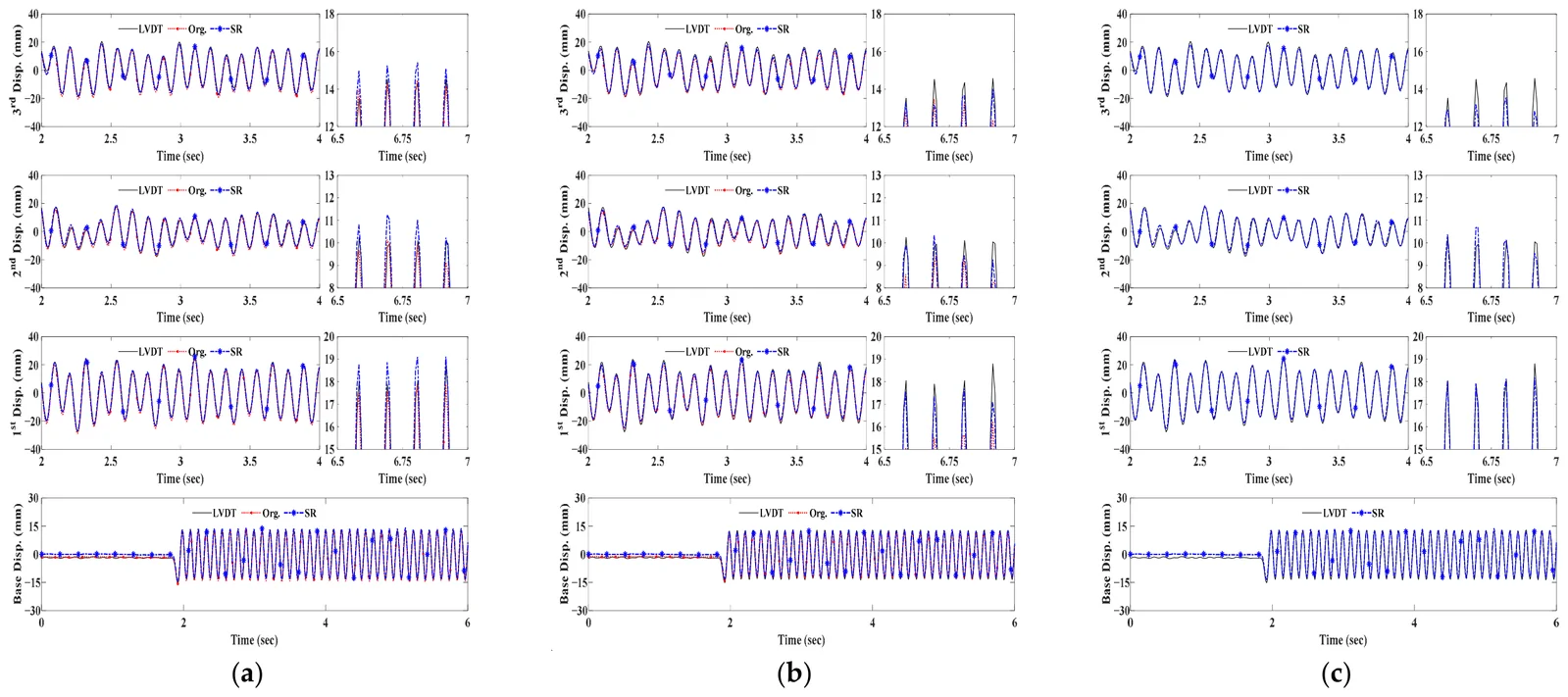
Displacement measurement comparison at 40 m depending on targets. (**a**) KZF using artificial target. (**b**) KZF using bolt connection. (**c**) KZF using slab cross-section.

Representative long-range displacement responses are presented in Figure 12 and Figure 13. At extended measurement distances, the RSR-based measurements followed the LVDT reference more closely than the measurements obtained from the original images, exhibiting reduced waveform distortion and more stable peak estimation. Although similar tendencies had been previously observed under normal illumination, the improvement produced by RSR became much more significant under low illumination because the original image quality was more severely degraded. In cases where the original low-light images produced unstable or practically unusable displacement responses, the RSR-restored images enabled continuous tracking and yielded time-history responses that were qualitatively consistent with the LVDT measurements. These observations indicate that the structural information recovered by RSR directly contributes to the restoration of measurement stability at long distances under poor lighting.

The quantitative error comparison is summarized in Table 5. For the bolt-connection target at 30 m under normal illumination, the RMSE decreased from 0.79 mm for the original image to 0.70 mm for the RSR image. A more substantial improvement was observed at 40 m, where the RMSE of the KZF-based measurement decreased from 1.20 mm to 0.87 mm after applying RSR, corresponding to an improvement of approximately 27.5%. More importantly, under low illumination at 40 m, the slab cross-section became measurable after RSR was applied, yielding RMSE values of 0.92 mm for STC and 0.74 mm for KZF. This result is particularly meaningful because it demonstrates that SR can convert previously unreliable or nearly infeasible low-light measurement conditions into usable data. The lower RMSE obtained by KZF further indicates that the restored texture and boundary information were more effectively utilized by the KAZE-based approach than by the Shi–Tomasi-based approach.

These findings clearly demonstrate that SR can mitigate the coupled limitations of low illumination and low spatial resolution in long-range vision-based displacement measurement. Whereas the baseline results in Section 3.1 showed that natural targets rapidly lost tracking reliability as distance increased under low illumination, the results in this section confirm that SR can recover sufficient structural detail to partially restore measurement capability. In practical terms, the application of RSR effectively extends the usable measurement range of the vision sensor under low-light conditions, particularly for natural targets that would otherwise approach the detection limit at long distances. It should be noted that this improvement is not simply a consequence of the higher image resolution. In Section 3.2, the bicubic and RealSR images were compared at an identical pixel resolution, and only the RealSR images restored the structural detail required for stable tracking; the improvement observed at extended distances is therefore attributed to the restored structural information rather than to the finer pixel grid alone.

To verify the dynamic accuracy in the frequency domain, the frequency content of the displacement responses reported in Figure 12 and Figure 13 was examined over the time window shown. All response curves exhibited a dominant frequency of 9.0 Hz, consistent with the applied harmonic excitation, and the 9 Hz amplitudes of the LVDT and the vision-based measurements agreed to within a few percent. The agreement was closest for the RealSR-based measurements, which reproduced the LVDT amplitude more closely than the original images, particularly for the natural targets at 40 m.

Taken together, the results presented in this section indicate that the degradation in vision-based displacement measurement under low illumination is primarily attributable to the loss of structurally meaningful image information caused by reduced brightness, increased noise, and insufficient spatial resolution. This effect became more pronounced as the measurement distance increased and was especially severe for natural targets, whose detectability depended on weak and non-uniform surface texture rather than deliberately designed patterns. In comparison with the previously reported normal-illumination results, the present low-light experiments clearly demonstrate that poor lighting not only increases measurement error, but also accelerates the loss of tracking stability as the target becomes smaller in the image plane.

Within this context, the application of super-resolution was shown to be effective not simply as a visual enhancement technique, but as a practical means of restoring target-specific structural details that are essential for reliable feature extraction and displacement tracking. Among the examined combinations, the RealSR-based restoration consistently provided the most meaningful improvement, and its benefit became particularly evident when combined with the KAZE-based tracking approach under long-range, low-illumination conditions. The results therefore support the central premise of this study, namely that super-resolution can compensate, at least in part, for the coupled limitations of low illumination and low spatial resolution, thereby extending the practical applicability of vision sensors for structural displacement monitoring. These findings provide the basis for the concluding remarks of this study, in which the effectiveness, practical significance, and future applicability of the proposed framework are further discussed.

## 4. Conclusions

This study investigated the displacement measurement performance of a vision sensor under low-illumination conditions and evaluated how illumination degradation affects measurement accuracy and effective measurement distance through comparison with previously reported normal-illumination results. In addition, a super-resolution-based image restoration strategy was introduced to mitigate the image degradation observed under low-light, long-range conditions, and its effectiveness was experimentally examined. To this end, Shi–Tomasi- and KAZE-based feature extraction and tracking were applied to one artificial target and two natural targets, and the resulting displacement measurements were quantitatively evaluated against the reference data acquired from an LVDT.

The results showed that low illumination significantly degraded the overall performance of vision-based displacement measurement through the combined effects of reduced brightness, increased noise, blurred boundaries, and the loss of local texture information. This degradation became more pronounced as the measurement distance increased and was particularly severe for natural targets such as the bolt connection and slab cross-section, which depend on surface texture and local edge information rather than intentionally designed high-contrast patterns. In comparison with the normal-illumination benchmark, the low-light condition caused a more rapid decline in both feature detectability and tracking stability, and the performance gap became increasingly evident at longer distances.

In the comparison between feature detection approaches, the KAZE-based approach generally outperformed the Shi–Tomasi-based approach. The KAZE detector retained a larger number of valid feature points under low illumination and long-range conditions, and the corresponding KZF-based displacement measurements exhibited better waveform reproducibility and lower error levels than the STC-based results. These findings indicate that under degraded imaging conditions, measurement performance depends not only on the number of extracted feature points, but also on the quality and spatial distinctiveness of the structural information available for tracking.

The application of super-resolution-based image restoration further demonstrated that RealSR was more effective than simple interpolation-based enlargement in improving both feature extraction and displacement measurement performance. After super-resolution was applied, blurred boundaries and weakened texture information in low-light images were partially recovered, leading to improved feature stability and tracking reliability. In particular, reductions in RMSE were observed under low illumination at 10 and 20 m, and under long-range conditions, natural targets that were unstable or nearly unmeasurable in the original images became measurable after super-resolution-based restoration. These results indicate that super-resolution is not merely a visual enhancement technique, but a practical preprocessing strategy that can substantially improve the effectiveness of vision-based measurement under low illumination.

The benefit of super-resolution became even more evident at long measurement distances. As demonstrated in Section 3, the original low-light images exhibited a sharp reduction in usable feature points and a corresponding deterioration in displacement response stability as the distance increased. In contrast, the super-resolved images provided more reliable feature sets and improved agreement with the LVDT reference response. As a result, the proposed framework showed the potential to extend the effective measurement range of low-light vision-based monitoring from conditions previously limited to approximately 30 m toward 40 m, particularly for natural targets. This finding suggests that the proposed method contributes not only to the reduction in measurement error, but also to the practical expansion of the operational range of vision sensors.

Overall, this study experimentally demonstrated that the degradation in vision-based displacement measurement under low illumination originates primarily from the loss of structurally meaningful image information, and that this limitation can be effectively alleviated through super-resolution-based restoration. Among the examined combinations, the integration of RealSR and the KAZE-based approach provided the most stable and accurate performance, suggesting that it is a promising solution for long-range structural displacement monitoring under poor lighting conditions. Therefore, the proposed framework can serve as a practical means of improving the applicability of vision sensors in field environments where lighting conditions are limited, such as nighttime measurements, tunnel interiors, and the undersides of bridges.

Nevertheless, this study also has several limitations. The experiments were conducted under a limited range of illumination levels, measurement distances, target types, and laboratory conditions. In real field environments, time-varying illumination, atmospheric turbulence, camera vibration, lens distortion, external light interference, and surface contamination or aging of the structure may simultaneously affect the image quality and measurement performance. In addition, the restoration performance may vary depending on the type of super-resolution model and the characteristics of its training data. Therefore, future research should validate the robustness of the proposed framework under a wider range of low-light conditions and on full-scale structures. Further investigation is also needed regarding real-time implementation, the use of alternative feature detectors and tracking algorithms, and the trade-off between computational cost and measurement improvement. In addition, because the upscaled images used in Section 3.3 were generated at twice the resolution of the original images, the tracking window covered a smaller physical area in the upscaled images than in the original ones; the contribution of image restoration, as distinct from that of the finer pixel grid, was therefore assessed through the resolution-controlled comparison of the bicubic and RealSR images reported in Section 3.2. In addition, the comparison in this study is limited to interpolation-based and super-resolution-based upscaling; other preprocessing strategies such as denoising and contrast enhancement, which modify the intensity content of the image rather than its spatial resolution, were not compared.

Future work will therefore proceed in several directions. First, the proposed framework should be validated under time-varying and non-uniform illumination, on full-scale structures and outdoor bridge sites, and under adverse environmental effects such as atmospheric turbulence, camera vibration, and lens distortion. Second, because the present results indicate that the benefit of super-resolution depends on the target type and on the feature detector, a broader comparative study of alternative detectors (e.g., SIFT, ORB, and AKAZE) and trackers, together with the incorporation of deep-learning-based matching, is required. Third, the computational cost of the super-resolution stage should be assessed with respect to real-time implementation, including GPU acceleration and its trade-off with measurement accuracy. Fourth, since GAN-based super-resolution may synthesize high-frequency structures that are not physically present, future studies should quantify and suppress such hallucination effects, for example, by combining the restored image with a fidelity-preserving image or by adopting artifact-aware super-resolution training. Finally, the framework should be extended to multi-target and multi-camera monitoring, and its generality should be examined with other super-resolution models and training datasets.

In conclusion, this study quantitatively identified the limitations of vision-sensor-based structural displacement measurement under low-illumination conditions and demonstrated that super-resolution-based image restoration can improve both measurement accuracy and effective measurement distance. These findings provide a useful basis for extending the field applicability of vision-based structural health monitoring systems and are expected to contribute to the advancement of image-based measurement techniques for challenging low-light environments.

## Figures and Tables

**Table 1:** Number of features detected.

Illumination	Feature Type	Target Type	Measurement Distance (m)			
			10	20	30	40
Normal (450 lux)	Corner points (no.)	A ^1^	90	82	41	29
		B ^2^	25	9	4	2
		S ^3^	46	9	-	-
	KAZE features (no.)	A	288	100	54	34
		B	36	16	10	5
		S	55	16	-	-
Low (120 lux)	Corner points (no.)	A	101	100	39	28
		B	29	8	3	2
		S	52	11	-	-
	KAZE features (no.)	A	289	99	46	31
		B	35	14	8	5
		S	54	16	-	-

^1^ Artificial target, ^2^ bolt connection, and ^3^ slab cross-section.

**Table 2:** Error quantification of feature point-based displacement measurement system depending on the measurement distance changes.

Illumination	Disp. Measurement	Error	Target Type	Measurement Distance (m)			
				10	20	30	40
Normal (450 lux)	STC	RMSE (mm)	A ^1^	0.62	0.67	0.70	0.75
			B ^2^	0.65	0.69	0.79	-
			S ^3^	0.66	0.67	-	-
		RPE (%)	A	1.38	1.45	1.52	1.58
			B	1.74	1.85	−4.30	-
			S	1.55	1.57	-	-
	KZF	RMSE (mm)	A	0.61	0.63	0.66	0.74
			B	0.63	0.65	0.76	1.20
			S	0.66	0.68	-	-
		RPE (%)	A	1.40	1.43	1.50	1.56
			B	1.72	1.81	−4.19	−10.22
			S	1.56	1.59	-	-
Low (120 lux)	STC	RMSE (mm)	A	0.64	0.76	0.81	0.86
			B	0.66	0.78	0.87	-
			S	0.65	0.76	-	-
		RPE (%)	A	1.38	1.52	1.65	1.73
			B	1.77	1.91	−4.31	-
			S	1.56	1.67	-	-
	KZF	RMSE (mm)	A	0.64	0.73	0.79	0.85
			B	0.65	0.78	0.83	1.24
			S	0.65	0.75	-	-
		RPE (%)	A	1.36	1.50	1.63	1.71
			B	1.75	1.89	−4.26	−10.23
			S	1.56	1.64	-	-

^1^ Artificial target, ^2^ bolt connection, and ^3^ slab cross-section.

**Table 3:** Quantitative result of image restoration.

Illumination	Measurement Dist. (m)	PSNR (dB) ↑		SSIM ↑		LPIPS ↓	
		BC ^1^	RSR ^2^	BC	RSR	BC	RSR
Normal (450 lux)	10	32.11	32.34	0.91	0.90	0.29	0.21
	20	35.85	32.92	0.95	0.87	0.22	0.22
Low (120 lux)	10	35.51	35.08	0.91	0.91	0.33	0.27
	20	40.70	36.22	0.96	0.89	0.28	0.26

↑ and ↓ mean higher value or lower value is desired. ^1^ Bicubic upsampling and ^2^ RealSR.

**Table 4:** Error quantitative analysis. The results are reported for the artificial target (A), the bolt connection (B), and the slab cross-section (S) at measurement distances of 10 m and 20 m, under both normal (450 lux) and low (120 lux) illumination, and for the original (Org.), bicubic (BC), and RealSR (RSR) images.

Illumination	Disp. Measurement	Error	Target Type	Measurement Distance (m)					
				10			20		
				Org.	BC ^4^	RSR ^5^	Org.	BC	RSR
Normal (450 lux)	STC	RMSE (mm)	A ^1^	0.62	0.72	0.70	0.67	0.68	0.64
			B ^2^	0.65	0.76	0.73	0.69	0.72	0.68
			S ^3^	0.66	0.74	0.70	0.67	0.70	0.65
		RPE (%)	A	1.38	1.59	1.43	1.45	1.53	1.37
			B	1.74	1.84	1.68	1.85	1.95	1.80
			S	1.55	1.79	1.63	1.57	1.77	1.61
	KZF	RMSE (mm)	A	0.61	0.69	0.69	0.63	0.59	0.59
			B	0.63	0.69	0.70	0.65	0.59	0.59
			S	0.66	0.70	0.71	0.68	0.60	0.60
		RPE (%)	A	1.43	1.61	1.44	1.43	1.63	1.47
			B	1.72	1.87	1.71	1.81	1.99	1.84
			S	1.55	1.78	1.62	1.59	1.66	1.50
Low (120 lux)	STC	RMSE (mm)	A	0.64	0.57	0.57	0.76	0.58	0.58
			B	0.66	0.58	0.58	0.78	0.58	0.58
			S	0.65	0.59	0.60	0.76	0.79	0.59
		RPE (%)	A	1.38	1.63	1.41	1.52	1.59	1.41
			B	1.77	1.97	1.75	1.91	2.00	1.70
			S	1.56	1.81	1.58	1.67	1.81	1.57
	KZF	RMSE (mm)	A	0.64	0.58	0.58	0.73	0.59	0.59
			B	0.65	0.58	0.58	0.78	0.59	0.59
			S	0.65	0.57	0.57	0.75	0.61	0.61
		RPE (%)	A	1.36	1.61	1.38	1.50	1.67	1.40
			B	1.75	2.01	1.78	1.89	2.03	1.76
			S	1.56	1.73	1.51	1.64	1.71	1.57

^1^ Artificial target, ^2^ bolt connection, ^3^ slab cross-section, ^4^ bicubic upsampling, and ^5^ RealSR.

**Table 5:** Error quantification of the vision-based measurement system using upsampled images. The results are reported for the artificial target (A), the bolt connection (B), and the slab cross-section (S) at measurement distances of 30 m and 40 m, under both normal (450 lux) and low (120 lux) illumination, and for the original (Org.), bicubic (BC), and RealSR (RSR) images.

Illumination	Disp. Measurement	Error	Target Type	Measurement Distance (m)			
				30		40	
				Org.	RSR ^4^	Org.	RSR
Normal (450 lux)	STC	RMSE (mm)	A ^1^	0.70	0.67	0.75	0.72
			B ^2^	0.79	0.70	-	0.77
			S ^3^	-	0.68	-	0.85
		RPE (%)	A	1.52	1.44	1.58	1.54
			B	−4.30	1.96	-	−5.10
			S	-	1.74	-	−6.80
	KZF	RMSE (mm)	A	0.66	0.65	0.74	0.68
			B	0.76	0.65	1.20	0.87
			S	-	0.63	-	0.76
		RPE (%)	A	1.50	1.42	1.56	1.45
			B	−4.19	1.80	−10.22	−4.84
			S	-	1.58	-	1.61
Low (120 lux)	STC	RMSE (mm)	A	0.81	0.79	0.86	0.74
			B	0.87	0.82	-	0.86
			S	-	0.83	-	0.92
		RPE (%)	A	1.65	1.53	1.73	1.56
			B	−4.31	1.93	-	−6.05
			S	-	1.77	-	−7.29
	KZF	RMSE (mm)	A	0.79	0.64	0.85	0.70
			B	0.83	0.65	1.24	0.74
			S	-	0.66	-	0.74
		RPE (%)	A	1.63	1.40	1.71	1.56
			B	−4.26	1.79	−10.23	−6.95
			S	-	−4.22	-	−4.22

^1^ Artificial target, ^2^ bolt connection, ^3^ slab cross-section, ^4^ RealSR.

## Data Availability

The data presented in this study are available from the corresponding author upon reasonable request.
